# Sex Effects on Gene Expression in Lacrimal Glands of Mouse Models of Sjögren Syndrome

**DOI:** 10.1167/iovs.18-25772

**Published:** 2018-11

**Authors:** Sara Tellefsen, Mathias Kaurstad Morthen, Stephen M. Richards, Scott M. Lieberman, Raheleh Rahimi Darabad, Wendy R. Kam, David A. Sullivan

**Affiliations:** 1 Schepens Eye Research Institute of Massachusetts Eye and Ear, Boston, Massachusetts, United States; 2 Department of Medical Biochemistry, Oslo University Hospital/Faculty of Medicine, University of Oslo, Oslo, Norway; 3 Department of Genetics and Evolution, School of Biological Sciences, The University of Adelaide, Adelaide, Australia; 4 Stead Family Department of Pediatrics, Carver College of Medicine, University of Iowa, Iowa City, Iowa, United States; 5 Department of Clinical Anesthesia, Indiana University School of Medicine, Indianapolis, Indiana, United States; 6 Department of Ophthalmology, Harvard Medical School, Boston, Massachusetts, United States

**Keywords:** sex differences, Sjögren syndrome, lacrimal gland, gene expression, MRL/lpr-lpr/lpr mice, nonobese diabetic mice

## Abstract

**Purpose:**

Sjögren syndrome is an autoimmune disease that occurs primarily in women, and is associated with lacrimal gland inflammation and aqueous-deficient dry eye. We hypothesize that sex-associated differences in lacrimal gland gene expression are very important in promoting lymphocyte accumulation in this tissue and contribute to the onset, progression, and/or severity of the inflammatory disease process. To test our hypothesis, we explored the nature and extent of sex-related differences in gene expression in autoimmune lacrimal glands.

**Methods:**

Lacrimal glands were collected from age-matched, adult, male and female MRL/MpJ-Tnfrsf6^lpr^ (MRL/lpr) and nonobese diabetic/LtJ (NOD) mice. Glands were processed for the analysis of differentially expressed mRNAs by using CodeLink Bioarrays and Affymetrix GeneChips. Data were evaluated with bioinformatics and statistical software.

**Results:**

Our results show that sex significantly influences the expression of thousands of genes in lacrimal glands of MRL/lpr and NOD mice. The immune nature of this glandular response is very dependent on the Sjögren syndrome model. Lacrimal glands of female, as compared with male, MRL/lpr mice contain a significant increase in the expression of genes related to inflammatory responses, antigen processing, and chemokine pathways. In contrast, it is the lacrimal tissue of NOD males, and not females, that presents with a significantly greater expression of immune-related genes.

**Conclusions:**

These data support our hypothesis that sex-related differences in gene expression contribute to lacrimal gland disease in Sjögren syndrome. Our findings also suggest that factors in the lacrimal gland microenvironment are critically important in mediating these sex-associated immune effects.

Sjögren syndrome is an autoimmune disease often accompanied by chronic and extensive inflammation of the lacrimal glands.^1,2^ This lymphocyte infiltration may severely damage acinar and ductal epithelial cell function, resulting in a significantly diminished output of aqueous tears.^1^ In consequence, Sjögren syndrome is a leading cause of aqueous-deficient dry eye disease.^1^

One of the most compelling features of Sjögren syndrome is that it affects predominantly females.^3–5^ In fact, female sex is a significant risk factor for the development of Sjögren syndrome, given that 93% of the patient population is female.^3–5^ This sexual dichotomy is frequently linked to fundamental sex-related differences in the immune system.^4,6,7^ Women have a more potent and competent systemic immune capability than men, and this heightened immunological activity is believed to contribute to the much greater incidence of many autoimmune diseases in females.^3,4,6,7^ Indeed, women constitute almost 80% of the 20 million people in the United States with autoimmune disease.^8^

We hypothesize that sex-associated differences in lacrimal gland gene expression are also very important in promoting lymphocyte accumulation in this tissue and contribute to the onset, progression, and/or severity of the inflammatory disease process. Consistent with this hypothesis is our discovery that the expression of a number of proto-oncogenes and apoptotic genes are significantly increased in the inflamed lacrimal tissues of female, as compared with male, MRL/lpr mice.^9^

To continue to test our hypotheses, we sought to explore further the nature and extent of sex-related differences in gene expression in autoimmune lacrimal glands. Toward that end, we examined and compared the gene expression in lacrimal glands of female and male MRL/MpJ-Tnfrsf6^lpr^ (MRL/lpr) and nonobese diabetic/LtJ (NOD) mice, respectively. The extent of lacrimal and salivary gland inflammation in MRL/lpr mice is, as in humans, far greater in females as compared with males.^10^ In contrast, although the salivary gland immunopathology in NOD mice is more extensive in females, the magnitude of lacrimal gland inflammation is far worse in NOD males (Toda I, et al. *IOVS* 1997;34:ARVO Abstract 434).^10,11^ We believe that this differential autoimmune expression in lacrimal glands of MRL/lpr and NOD mice reflects, in large part, the influence of local tissue, as compared with systemic, factors.

## Materials and Methods

### Animals and Tissue Collections

Adult male and female MRL/lpr and NOD mice were obtained from the Jackson Laboratories (Bar Harbor, ME, USA). Mice (*n* = 15 to 18/sex/strain) were housed in constant temperature rooms with fixed light/dark intervals of 12 hours' length. When indicated, mice were killed by CO_2_ inhalation and exorbital lacrimal glands were removed for molecular biological procedures. Lacrimal gland samples were prepared by combining tissues from five to six mice/sex/group. Three different sample preparations were made for each tissue/sex/group and then processed for the analysis of gene expression.

All research experiments with mice were approved by the Institutional Animal Care and Use Committee of The Schepens Eye Research Institute and adhered to the ARVO Statement for the Use of Animals in Ophthalmic and Vision Research.

### Molecular Biological Procedures

Total RNA was extracted from lacrimal glands by using TRIzol reagent (Invitrogen Corp., Carlsbad, CA, USA) and purified with RNAqueous spin columns (Ambion, Austin, TX, USA). The lacrimal gland RNA samples were treated with RNase-free DNase (Invitrogen), analyzed spectrophotometrically at 260 nm to determine concentration, and evaluated with an RNA 6000 Nano LabChip and an Agilent 2100 Bioanalyzer (Agilent Technologies, Palo Alto, CA, USA) to confirm RNA integrity. The RNA samples were then stored at −80°C until further processing.

Gene expression was examined by the use of two procedures. One involved the processing of RNA samples for hybridization to CodeLink UniSet Mouse 20K I Bioarrays (*n* ∼ 20,000 genes/array; Amersham Biosciences/GE Healthcare, Piscataway, NJ, USA), according to detailed methods.^12^ cDNA was synthesized from RNA (2 μg) with a CodeLink Expression Assay Reagent Kit (Amersham) and purified with a QIAquick purification kit (Qiagen, Valencia, CA, USA). Samples were dried, and cRNA was generated with a CodeLink Expression Assay Reagent Kit (Amersham), recovered with an RNeasy kit (Qiagen) and quantitated with an UV spectrophotometer. Fragmented, biotin-labeled cRNA was then incubated and shaken at 300 rpm on a CodeLink Bioarray at 37°C for 18 hours. After this time period, the Bioarray was washed, exposed to streptavidin-Alexa 647, and scanned by using ScanArray Express software and a ScanArray Express HT scanner (Packard BioScience, Meriden, CT, USA) with the laser set at 635 nm, laser power at 100%, and photomultiplier tube voltage at 60%. Scanned image files were evaluated by using CodeLink image and data analysis software (Amersham), which yielded both raw and normalized hybridization signal intensities for each array spot. The intensities of the approximately 20,000 spots on the Bioarray image were standardized to a median of 1. Normalized data, with signal intensities greater than 0.50, were analyzed with bioinformatic software (Geospiza, Seattle, WA, USA). This sophisticated software also created gene ontology, Kyoto Encyclopedia of Genes and Genomes (KEGG) pathway and *z*-score reports. The ontologies encompassed biological processes, molecular functions, and cellular components and were organized according to the recommended guidelines of the Gene Ontology Consortium (http://www.geneontology.org/GO.doc.html).^13^

The second method to examine differential gene expression involved the hybridization of each cRNA (20 μg) sample to a GeneChip Mouse Genome 430A 2.0 Array (Affymetrix, Santa Clara, CA, USA) according to the manufacturer's protocol. Reagents for the fragmentation and hybridization steps were from a GeneChip HT One-Cycle Target Labeling and Control Kit, and materials for the washing and staining steps came from a GeneChip HWS kit (Affymetrix). Hybridized GeneChips were scanned with an Affymetrix Model 700 Scanner and expression data files were created from array images by using Affymetrix Microarray Suite 4.0 software. GeneChip data were standardized by choosing the default scaling in Affymetrix GeneChip Operating Software, which yields a trimmed mean intensity of 500 for each GeneChip microarray. Normalized data with a quality value of 1.0 were then analyzed with Geospiza GeneSifter software (Geospiza).

Counts of unique mappings of probes to gene identifications in the CodeLink and Affymetrix arrays showed that there were 15,711 and 13,265 unique genes, respectively, in these arrays. Analysis of the intersection of these lists demonstrated that there was an overlap of 11,299 genes.

Gene expression data were examined without log transformation and statistical analyses of these data were performed with Student's *t*-test (two-tailed, unpaired) by using the GeneSifter software. Our statistical approach was not tailored for multiple comparisons. Genes that were expressed in the same direction in different groups were identified by using GenBank accession numbers and an intersector program (Geospiza). Data used for these CodeLink and Affymetrix arrays are accessible for free download through the National Center for Biotechnology Information's Gene Expression Omnibus via series accession number GSE5876.

## Results

### Influence of Sex on Gene Expression in Lacrimal Glands of MRL/lpr and NOD Mice

To determine the influence of sex on gene expression in lacrimal glands of autoimmune mice, tissues were obtained after disease onset^10^ from MRL/lpr (*n* = 18 mice/sex; age = 19.8 ± 0.3 weeks old) and NOD (*n* = 15 mice/sex; age = 21.4 weeks old) mice. Glands were pooled according to sex and group (*n* = 10–12 glands/sex/sample; *n* = 3 samples/sex/group), processed for the isolation of total RNA, and examined for differentially expressed mRNAs by using CodeLink Bioarrays and Affymetrix GeneChips. Microarray data were analyzed with Geospiza bioinformatics software.

Our findings demonstrate that sex has a significant impact on the expression of thousands of genes in lacrimal glands of MRL/lpr and NOD mice (Table 1). Non-sex chromosome genes with the greatest differences in terms of expression ratios in MRL/lpr mice are shown in Table 2. Genes, such as pancreatic lipase-related protein 1, asialoglycoprotein receptor, S100 calcium-binding proteins A8 and A9, and growth differentiation factor 5, were increased in females, and lymphocyte antigen 6 complex, locus F and cytochrome P450, family 2, subfamily j, polypeptide 13 in were higher in males, and the results were similar with both CodeLink and Affymetrix microarrays.

**Table 1:**
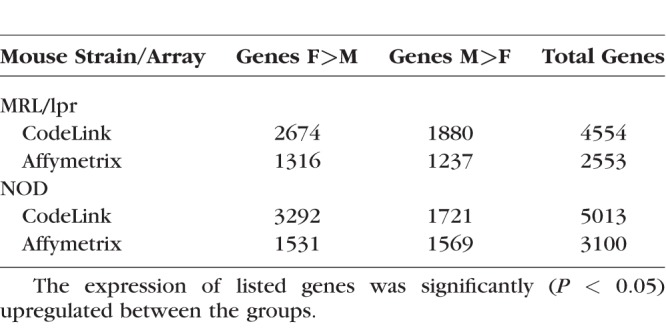
Number of Genes With Significant, Sex-Related Differences in Expression in Lacrimal Glands of MRL/lpr and NOD Mice

**Table 2:**
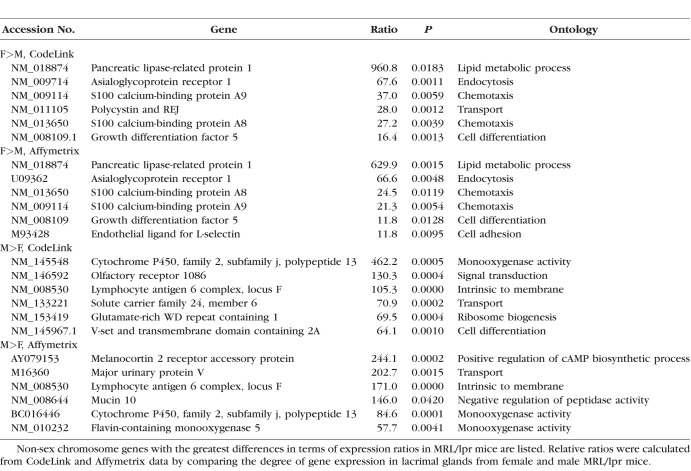
Influence of Sex on Gene Expression in Lacrimal Glands of MRL/lpr Mice

Additional genes of interest included that for cathepsin S, which is significantly increased in the tears of Sjögren syndrome patients,^14^ and is more highly expressed in lacrimal tissues of female MRL/lpr mice (CodeLink = 2.85-fold; Affymetrix = 3.03-fold). Also notable were the increased expression of X-chromosome genes, such as X inactive specific transcript (Xist) (CodeLink = 32.0-fold), domesticus antisense RNA from the Xist locus (Affymetrix = 27.7-fold), and moesin (Affymetrix = 3.45-fold) in females, and the X (androgen receptor; CodeLink = 1.7-fold) and Y (eukaryotic translation initiation factor 2, subunit 3; CodeLink = 60.1-fold; Affymetrix = 205.2-fold) chromosome genes in males.

Genes with many of the highest expression differences in terms of ratios in NOD mice are shown in Table 3. Some of these genes (e.g., female [F] > male [M], pancreatic lipase-related protein 1 and asialoglycoprotein receptor; M>F, cytochrome P450, family 2, subfamily j, polypeptide 13, and neuromedin U) showed analogous degrees of difference in both the CodeLink and Affymetrix microarrays. Elevated levels of Y chromosome genes, including gene eukaryotic translation initiation factor 2, subunit 3 (CodeLink = 48.8-fold; Affymetrix = 10.1-fold) and DEAD box polypeptide 3 (Affymetrix = 115.1-fold) were also found in lacrimal glands of males, whereas the expression of the X-chromosome gene, androgen receptor (Affymetrix = 3.06-fold), was greater in female lacrimal tissues. In contrast to the results with MRL/lpr mice, the expression of cathepsin S (CodeLink = 3.85-fold; Affymetrix = 6.06-fold) and the X-linked gene moesin (Affymetrix = 6.32-fold) were significantly higher in male lacrimal glands, as compared with those of females.

**Table 3:**
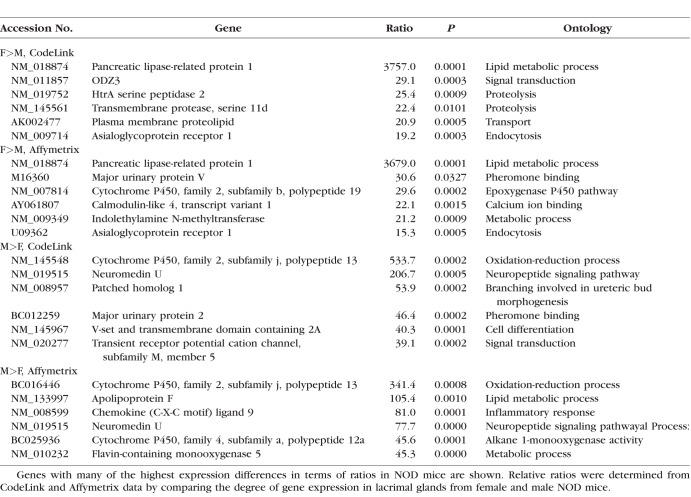
Effect of Sex on Gene Expression in Lacrimal Glands of NOD Mice

Most of the lacrimal gland genes in MRL/lpr and NOD female and male mice, respectively, which were identified as differentially expressed by the CodeLink and Affymetrix microarrays, were unique to each platform. As shown in Table 4, relatively few genes displaying sex-related differences were expressed by both microarrays. These findings are consistent with our previous investigations,^15–17^ as well as those of others,^18–21^ which discovered little agreement between CodeLink and Affymetrix microarrays in the detection of differential gene expression. Although these platforms seem to measure different things,^20^ most gene expression changes revealed by each of the platforms are thought to be biologically correct.^19,20^

**Table 4:**
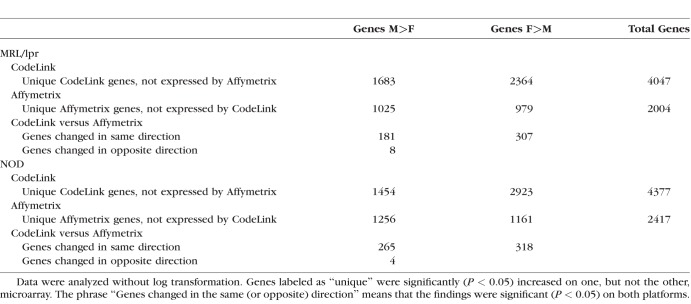
Comparison of Gene Expression Data Between CodeLink and Affymetrix Microarrays

Comparison of gene expression between the inflamed lacrimal glands of MRL/lpr (F>M) and NOD (M>F) mice showed that 465 genes were common (CodeLink). The alternate comparison (i.e., MRL/lpr, M>F; NOD, F>M) revealed 187 genes in common (CodeLink).

### Impact of Autoimmune Disease on Immune-related Biological Process, Molecular Function, and Cellular Component Ontologies in Lacrimal Glands of MRL/lpr Female and NOD Male Mice

Autoimmune disease had a dramatic impact on the expression of numerous immune-related gene ontologies in the lacrimal glands of female MRL/lpr and male NOD mice. Many of these ontologies were identified by both CodeLink and Affymetrix platforms.

As shown in Tables 5 and 6, the expression of immune-related ontologies in lacrimal tissues of female MRL/lpr and male NOD mice was significantly increased in all three major gene function areas, including biological processes (e.g., inflammatory response), molecular functions (e.g., chemokine activity), and cellular components (e.g., major histocompatibility complex [MHC] protein complex). These aspects, as defined by the Gene Ontology Consortium (http://www.geneontology.org/page/ontology-documentation), address the biological programs accomplished by multiple molecular activities (i.e., biological processes), the molecular-level activities performed by gene products (i.e., molecular functions), and the locations relative to cellular structures in which a gene product performs a function (i.e., cellular components).

**Table 5:**
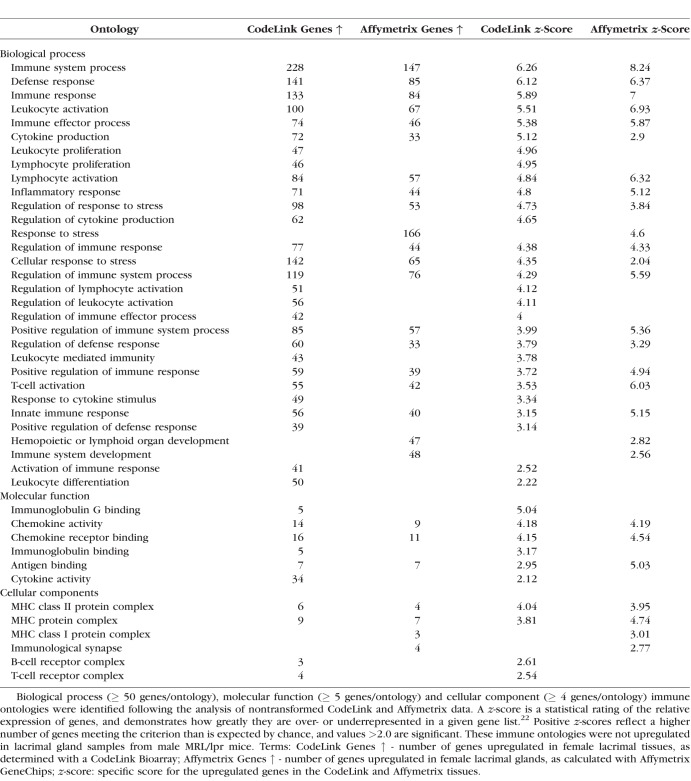
Immune Gene Ontologies Upregulated in Lacrimal Glands of Female MRL/lpr Mice

**Table 6:**
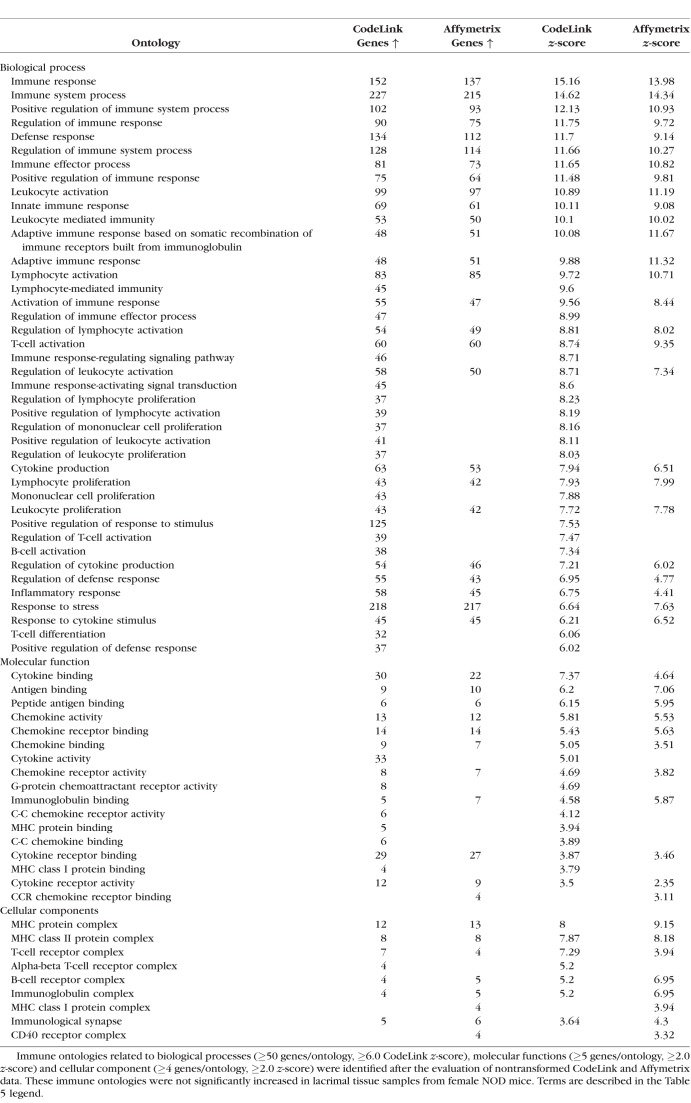
Immune Gene Ontologies Significantly Increased in Lacrimal Glands of Male NOD Mice

An example of the degree of autoimmune influence was demonstrated by analysis of biological process ontologies in male NOD lacrimal glands, which showed that 41 of the 53 most highly significant ontologies (≥ 50 genes/ontology; *z*-score ≥ 6.0) were all immune-related. One such ontology, inflammatory response, displayed a significant increase in multiple inflammatory genes by both CodeLink and Affymetrix microarrays in female MRL/lpr (Table 7) and male NOD (Table 8) mouse lacrimal tissues. Twenty-six of these inflammatory genes were the same in both female MRL/lpr and male NOD mice.

**Table 7:**
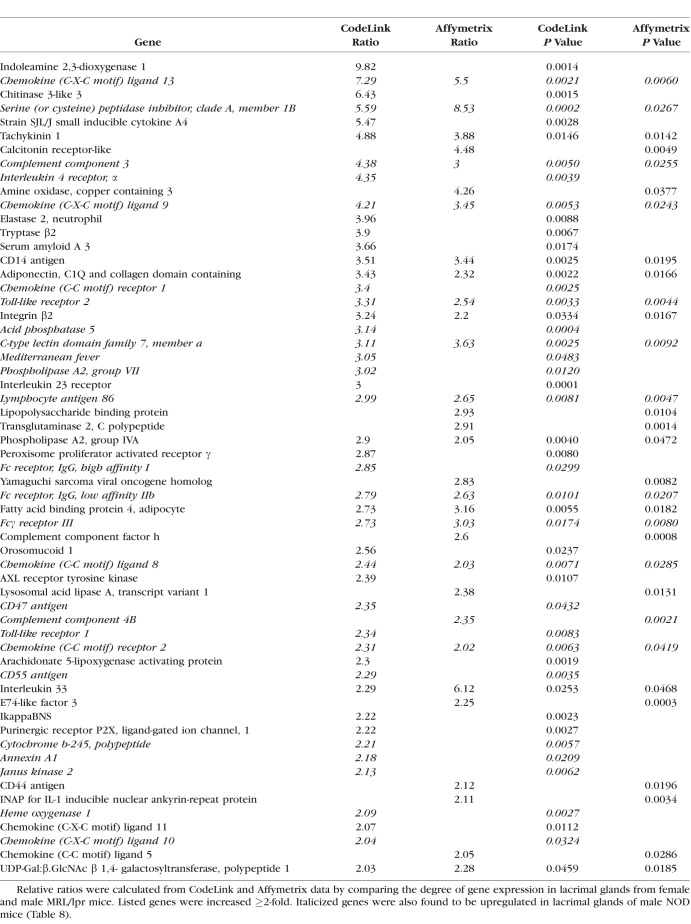
Increased Expression of Genes in Inflammatory Response Ontology in Lacrimal Glands From Female MRL/lpr Mice

**Table 8:**
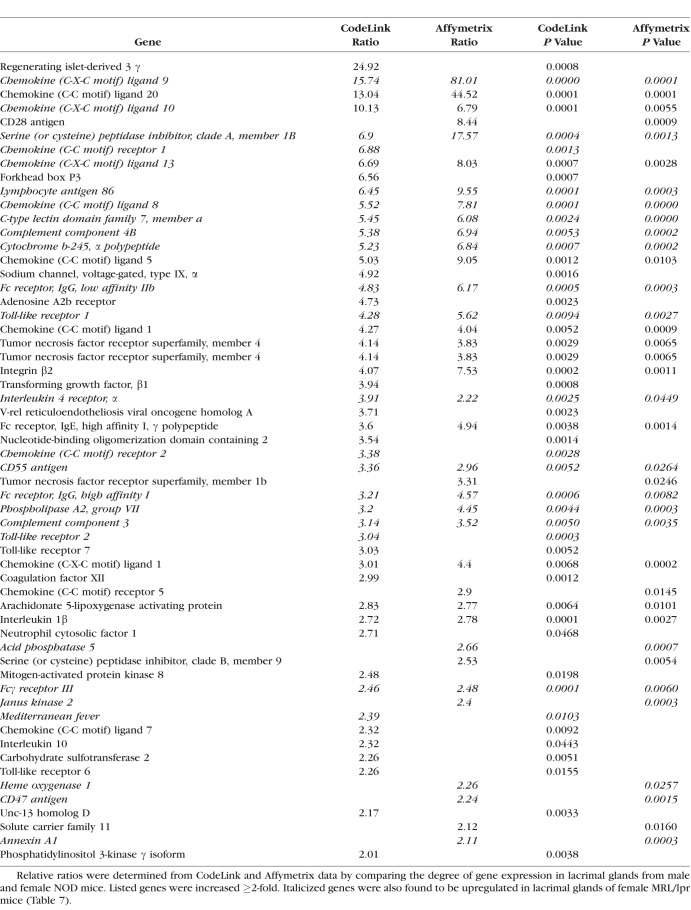
Increased Expression of Genes in Inflammatory Response Ontology in Lacrimal Glands From Male NOD Mice

### Effects of Autoimmune Disease on Immune-related KEGG Pathways in Lacrimal Glands of MRL/lpr Female and NOD Male Mice

Lacrimal gland samples from female MRL/lpr and male NOD mice also showed a significant increase in the expression of immune-related KEGG pathways (Tables 9 and 10). These included pathways related to antigen processing (Tables 11 and 12), chemokines (Tables 13 and 14), and Fcγ R-mediated phagocytosis (Table 10), as well as those linked to type 1 diabetes mellitus and systemic lupus erythematosus (SLE) (Tables 9 and 10). Inflammation in these tissues also significantly enhanced the expression of lysosome pathways (Affymetrix; MRL/lpr female, 19 genes upregulated ↑, *z*-score = 2.43; NOD male, 25 genes ↑, z-score = 3.68).

**Table 9:**
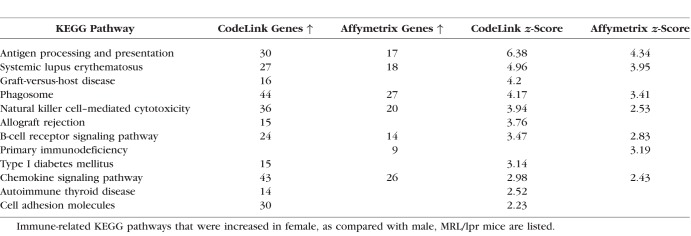
Immune KEGG Pathways Upregulated in Lacrimal Glands of Female MRL/lpr Mice

**Table 10:**
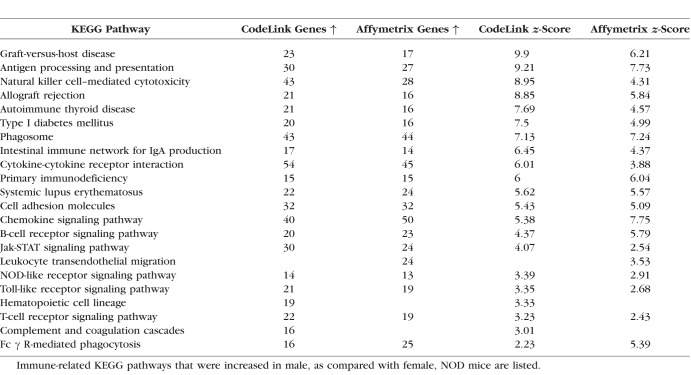
Immune KEGG Pathways Upregulated in Lacrimal Glands of Male NOD Mice

**Table 11:**
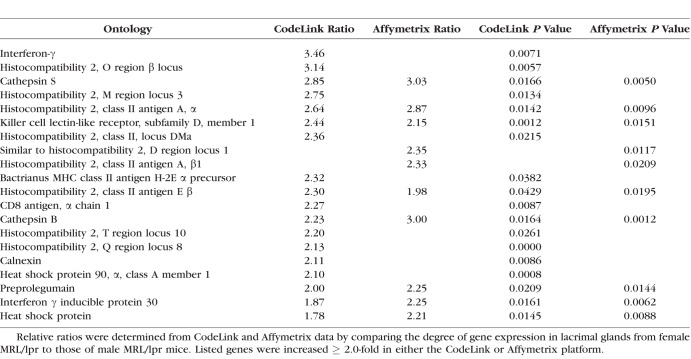
Upregulated Genes in the Antigen Processing KEGG Pathway in Lacrimal Glands From Female MRL/lpr Mice

**Table 12:**
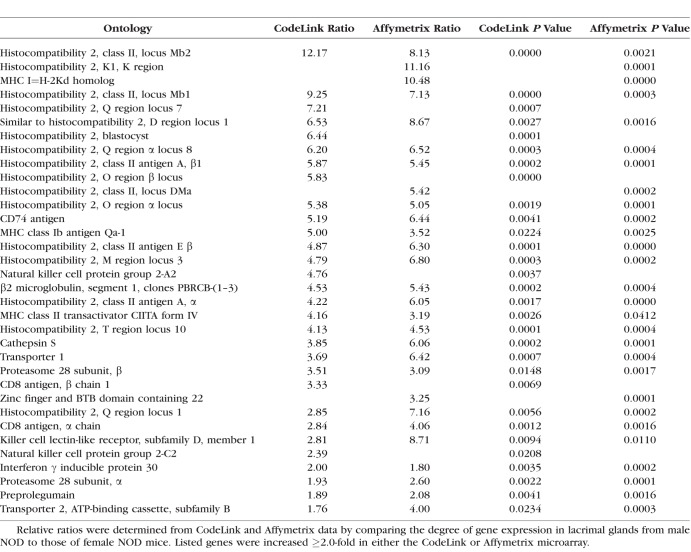
Upregulated Genes in the Antigen Processing KEGG Pathway in Lacrimal Glands From Male NOD Mice

**Table 13:**
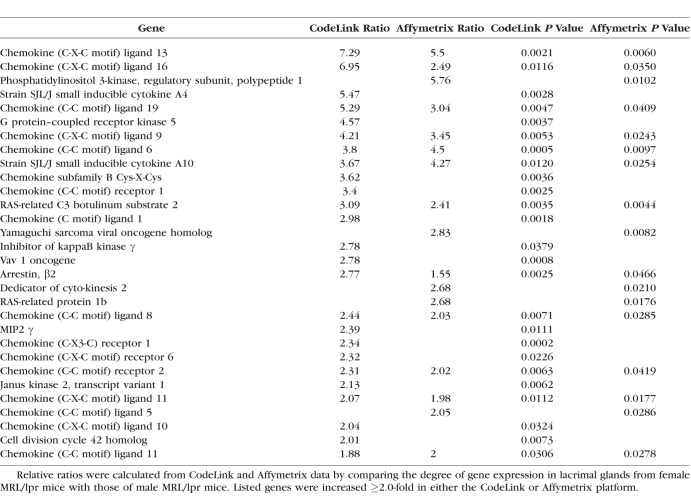
Heightened Gene Expression in the Chemokine KEGG Pathway in Lacrimal Glands of Female MRL/lpr Mice

**Table 14:**
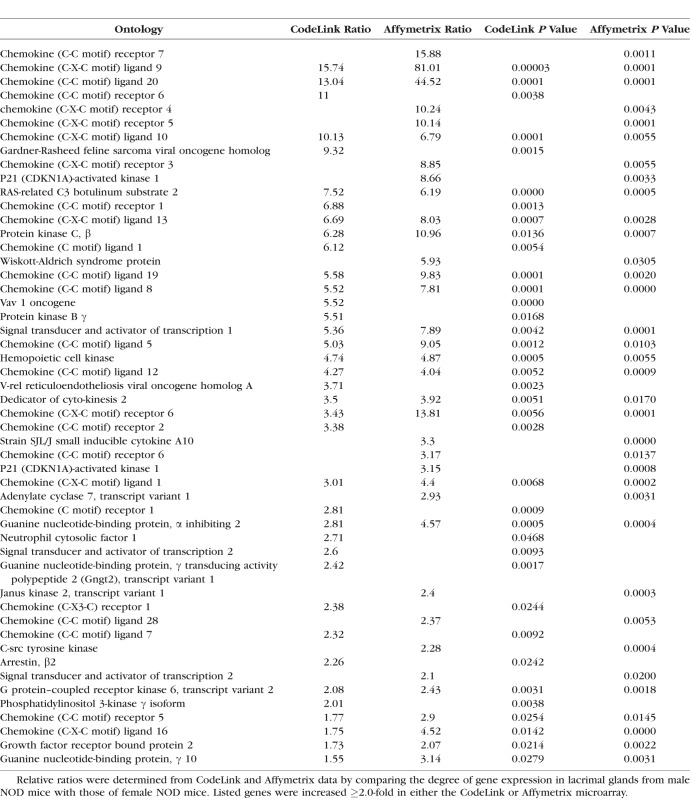
Increased Gene Expression in the Chemokine KEGG Pathway in Lacrimal Glands of Male NOD Mice

Of interest, an average of more 95% of the ribosome KEGG pathways were significantly increased in lacrimal glands of female MRL/lpr (CodeLink, 47 genes ↑, *z*-score = 9.64; Affymetrix, 17 genes ↑, *z*-score = 3.03) and NOD (CodeLink, 53 genes ↑, *z*-score = 10.78; Affymetrix, 59 genes ↑, *z*-score = 17.5) mice. Similarly, more that 81% of the proteasome KEGG pathways were significantly higher in lacrimal tissues of female MRL/lpr (Codelink, 22 genes ↑, *z*-score = 5.87; Affymetrix, 10 genes ↑, *z*-score = 2.77) and NOD (CodeLink, 21 genes ↑, *z*-score = 5.09; Affymetrix, 20 genes ↑, *z*-score = 7.4) mice.

## Discussion

Our results demonstrate that sex significantly influences the expression of thousands of genes in lacrimal glands of MRL/lpr and NOD mice. The nature of this sex-related expression, especially with regard to immune-associated genes, is very dependent on the specific mouse model of Sjögren syndrome. Lacrimal glands of female, as compared with those of male, MRL/lpr mice contain a significant increase in the expression of genes related to inflammatory responses, antigen processing, and chemokine pathways. In contrast, it is the lacrimal tissue of NOD males, and not NOD females, that presents with a significantly greater expression of immune-related genes. These findings support our hypothesis that sex-related differences in gene expression contribute to the onset, progression, and/or severity of the lacrimal gland inflammatory disease process. Our results also suggest that factors in the lacrimal gland microenvironment are critically important in mediating these sex-associated immune effects.

Our finding that significant sex-related differences exist in lacrimal gland gene expression in MRL/lpr and NOD mice was not unexpected. Significant, sex-associated differences are known to be present in the anatomy, physiology, and pathophysiology of the lacrimal gland. These differences are found in multiple species and include variations between males and females in the mean area and density of acinar complexes; the quantity of intercalated, intralobular, and interlobular ducts; the membrane contours, cytoplasmic appearance, vesicular content, and turnover of acinar epithelial cells; the position, size, and shape of acinar epithelial cell nuclei; the number of intranuclear inclusions; the prominence of nucleoli; the frequency of intercellular channels; the quantity of capillary endothelial pores; the expression of numerous genes; the synthesis, activity, phosphorylation, and affinity of many proteins, enzymes, and receptors; the population of lymphocytes; the expression of secretory immunity; the response to neural stimulation and drugs; the secretion of specific proteins; and the susceptibility to focal adenitis, fibrosis, atrophy, viral replication, and autoimmune disease.^5^

Three genes of particular interest that showed sexual dimorphism are those encoding ASGPR1, tripartite motif-containing 21 (TRIM21), and major urinary protein V (MUPV). First, expression of the ASGPR1 gene was many-fold greater in lacrimal glands of female, as compared with male, MRL/lpr mice. This receptor mediates the intracellular uptake of hepatitis C virus (HCV),^23^ thereby facilitating viral infection and increasing glandular inflammation.^23–25^ Chronic HCV infection, in turn, is linked to an enhanced prevalence of keratoconjunctivitis sicca^26^ and mimics the clinical manifestations of Sjögren syndrome.^24,25,27,28^ In addition, ASGPR is an autoantigenic target of both T and B cells.^29^ However, the ASGPR1 gene was also upregulated in lacrimal tissues of female NOD mice, which indicates that it is not a strain-independent inducer of inflammation.

Second, TRIM21, also known as Ro52/SSA, is a prominent antigen in Sjögren syndrome. Expression of TRIM21 was higher in lacrimal glands of female MRL/lpr mice (Affymetrix = 2.12-fold; CodeLink = 1.72-fold) and male NOD mice (Affymetrix = 2.71-fold; CodeLink = 1.45-fold). Antibodies targeting TRIM21/Ro52 are common in Sjögren syndrome patients and may be present years before diagnosis.^30^ Anti-TRIM21/Ro52 autoantibodies have also been detected in MRL/lpr and NOD mice.^31,32^ TRIM21/Ro52 is a ubiquitin E3 ligase that may be induced by interferons (type I or type II) and has immunomodulatory functions including regulation of proliferation and cell death, regulation of inflammatory cytokine production, and modulation of antiviral responses.^33–37^ Although these roles were largely described in immune cells, additional studies have detected an increase in TRIM21/Ro52 protein in salivary gland epithelial cell lines or salivary gland ductal epithelial cells from Sjögren syndrome patients.^38,39^ Expression of TRIM21/Ro52 has not, to our knowledge, been reported in lacrimal gland epithelial cells. Our findings of increased expression of TRIM21/Ro52 in lacrimal glands of MRL/lpr and NOD mice in the context of inflammation suggests this may contribute to the role of TRIM21/Ro52 as an autoantigen in Sjögren syndrome.

The third gene of particular interest is MUPV. This gene is one of the most highly upregulated genes in lacrimal glands of male MRL/lpr (202-fold) and female NOD (31-fold) mice. Hence, MUPV expression is inversely correlated with inflammation, and may possibly serve a protective function. Major urinary proteins are pheromone-binding lipocalins^40–43^ and implied effects include sexual attraction, aggression, hormone modulation, individual recognition, and spatial learning.^41,44,45^ Little is known about the relation of MUPV to sex and the immune system. However, considering that major urinary proteins function as pheromone-binding proteins, the pheromones themselves may play a role.

Such pheromones could be exocrine gland secreting peptides (ESPs), which are found in mice and exhibit sex-specific expression.^43,46–48^ ESP1 is male-specific, and its expression increases in response to androgen administration.^46^ In contrast, ESP36 is female-specific and is negatively regulated by androgen.^46^ Further, it has been suggested that the reception of ESPs in the vomeronasal system differs according to sex.^49^ The vomeronasal system is an accessory olfactory system, and pheromones also can be detected by the anatomically distinct main olfactory system.^46^ Of note, our CodeLink results showed that olfactory receptor 1086 is significantly upregulated in male lacrimal glands in MRL/lpr mice. This supports the concept of pheromone perception as an important factor in sexually dimorphic responses.^50^

Research has also provided evidence that the olfactory system may be inextricably linked to immunological function.^51^ For example, it has been shown that pheromone treatment suppresses hepatic inflammation in mice.^52^ Whether this effect has relevance to humans has not yet been determined, but it indicates that pheromone-sensing organs may have an underestimated value that warrants further investigation. Thus, it has been shown that patients with SLE have disturbances in olfactory function.^50^ The possible link between smell and autoimmunity may be due to gene location, considering that olfactory receptor gene clusters are located in close proximity to key loci of susceptibility for autoimmune disease, such as the MHC.^50^

In our study, a number of immune-related genes were upregulated in the lacrimal glands of female MRL/lpr and/or male NOD mice that may be important in the pathogenesis of Sjögren syndrome. These include the following: many interleukins, interferons, and their related proteins; the damage-associated molecular pattern proteins S100A8 and S100A9, which are expressed by neutrophils, monocytes, dendritic and epithelial cells, act as Toll-like receptor (TLR) ligands, and stimulate the production of multiple proinflammatory cytokines; myeloid differentiation primary response 88, which is used by most TLRs to activate nuclear factor-κB; B-cell linker, which regulates B-cell receptor signaling and development; the chemokines CXCL12, CXCL13, and CCL19, which promote the formation and perpetuation ectopic lymphoid structures; and the enzymes indoleamine 2,3-dioxygenase and kynurenine 3-monooxygenase, which ultimately may lead to immune system activation, inflammation, and the accumulation of potentially neurotoxic compounds.^53–57^

Numerous ontologies and KEGG pathways that were significantly upregulated in lacrimal tissues of female MRL/lpr and/or male NOD mice have also been linked to Sjögren syndrome. These ontologies encompass such immune system processes as antigen binding, T- and B-cell activation, signaling pathways, cytokine production, chemokine activity, and inflammatory responses, all of which appear to play a role in Sjögren syndrome pathogenesis.^4,58,59^ The increased expression of KEGG pathways related to lysosomes and Fcγ R-mediated phagocytosis was of particular interest, because they have been reported as the only pathways common to the development of the four autoimmune diseases type 1 diabetes mellitus, SLE, multiple sclerosis, and rheumatoid arthritis.^60^

A major question in our research is what triggers the sex-related inflammation in female MRL/lpr and male NOD lacrimal glands. There are a number of possibilities, some of which may be associated with sex chromosomes (i.e., X) and/or sex steroids (i.e., androgens).^5,53^ Thus, several recent studies suggest that the female prevalence of Sjögren syndrome is due to an X-chromosome dose effect, and that individuals with X-chromosome abnormalities like triple X syndrome (47 XXX) and Klinefelter syndrome (47 XXY) have an increased risk for developing the disease.^61–63^ In fact, attention has been drawn to X-chromosome vulnerability as a possible explanation for the high female prevalence of autoimmune diseases in general.^64–67^ Therefore, the genes located on the X-chromosome are especially intriguing. One such gene is moesin, which is significantly upregulated in female MRL/lpr and in male NOD lacrimal tissues. Moesin is a membrane organizing protein that plays a role in immunologic synapse formation, lymphoid cell regulation, and T regulatory cell (Treg) differentiation.^68,69^ In this last regard, there is evidence that a shift in the T helper cell 17 (Th17)/Treg balance toward the proinflammatory Th17 axis contributes to the development of Sjögren syndrome and other autoimmune disorders.^70–73^ The reasons for this shift are not completely known, but may be due, at least in part, to moesin activity and other microenvironmental stimuli.^52^

Another gene of particular interest is the X-chromosome–linked androgen receptor, the expression of which is increased in male MRL/lpr and female NOD lacrimal glands. Androgen receptors are members of the nuclear receptor superfamily of ligand-inducible transcription factors and appear to mediate almost all of the biological actions of androgens.^74,75^ Androgens, in turn, appear to be very important in Sjögren syndrome. For example, testosterone treatment of female MRL/lpr mice causes a dramatic suppression of the inflammation in, and a significant increase in the function of, the lacrimal gland.^5,76^ These effects are analogous to those found in humans, wherein topical or systemic androgen administration significantly decreases dry eye disease signs and symptoms, and stimulates tear flow, in patients with Sjögren syndrome.^5,76^ Indeed, androgen deficiency seems to be a risk factor for the development of lacrimal gland inflammation in women with Sjögren syndrome.^5,76^ In contrast, androgens induce lymphocyte infiltration into the lacrimal glands of NOD mice.^5,77,78^ This anomalous effect appears to be mediated through the lacrimal gland microenvironment,^11^ as well as male-specific factors that cause CD4(+) CD25(+) Foxp3(+) regulatory T-cell dysfunction.^78^ Further, this androgen response differs markedly from the androgen-induced decrease of inflammation in NOD salivary and pancreatic tissues.^11,79,80^

It is noteworthy that acinar and ductal epithelial cells contain the androgen receptors that are the target for androgen activity in lacrimal tissue.^81^ In addition, these cells are thought to be the primary cells involved in the initiation and perpetuation of glandular autoimmune reactivity in Sjögren syndrome.^82^ We hypothesize that this androgen-epithelial cell interaction induces the altered activity of specific genes in lacrimal glands, and leads to the reduction of pathological lesions and an improvement in glandular function in MRL/lpr, and the opposite effects in NOD, mice. Further research is required to test this hypothesis, and to identify those genes that may underlie the sex- and hormonal-regulation of the lacrimal gland in Sjögren syndrome.
